# The dilemma facing higher education and industry in tourism and hospitality

**DOI:** 10.1186/2193-1801-4-S2-P2

**Published:** 2015-11-27

**Authors:** Anthony Pui-keung Kong

**Affiliations:** Department of Hotel, Services and Tourism Studies, Hong Kong Institute of Vocational Education (Chai Wan), Hong Kong

## Background

Tourism and hospitality is a labour intensive industry. It is one of the four major pillars of the Hong Kong economy [1], and there is a huge demand for labour. However, Huyton [2] points out that hospitality and tourism graduates and students do not meet employers' expectations because of poor preparation and ability. Resolving this issue requires coordination of curriculum settings and industrial needs.

The research objectives of this study were to:

1. Identify perceptions and expectations among practitioners, educators and students in tourism and hospitality education.

2. Provide insight so schools can develop a suitable curriculum to train students to become qualified professionals.

## Methods

This study used a qualitative methodology of focus groups and in-depth interviews. Students and employers are considered to be two groups with first-hand experience who can play an advisory role and give critical comment on the applicability of current education to the industry. It is therefore necessary for the research design to take into account not just the perceptions of educators, but also those of students and employers. This triangulation is richly informative because it provides three different perspectives on the same issue.

The 24 interviewees for the study included 8 students, 8 employers and 8 educators. The 8 students were studying hospitality at Hong Kong Institution of Vocational Education, Hong Kong Polytechnic University and the Chinese University of Hong Kong. The employers, identified by purposive sampling, had been working in the hotel and tourism field for at least 5 years. The 8 educators were the programme leaders of Higher Diploma, Bachelor's and Master's degrees in hospitality.

## Results

*Finding 1*: Work experience is slightly more important to the students than to employers. The findings imply that work experience is not a 'must have', because employers do not expect new employees to know exactly how to accurately perform their tasks. The employers thought that it is time consuming to train new employees who have too much work experience because practices adopted in previous jobs need to be changed or adjusted to suit the new environment.

*Finding 2*: The employers demanded graduates with generic skills; academic education does not guarantee employment. Some employers did not prefer to recruit graduates from universities where the students study only theory and concepts of hospitality [3]. Jackling and De Lange [4] agreed that students who learnt generic skills in higher education had better employment opportunities.

*Finding 3*: Educators and employers blamed each other for the current situation. Educators commented that they had tried their best to come up with a well-rounded programme for their students but their workload could be very heavy so it was difficult to prepare teaching materials and also keep up with the changes in the industry [5]. At the same time, employers complained that graduates were not well-prepared and they lacked the required knowledge, skills and attitude.

## Conclusions

The students interviewed demonstrated a strong understanding of the importance of work experience, practical skills and academic education. Although they had a high expectation of employment in the industry, they seemed to be disappointed when pursuing a career in this field; they lacked passion and displayed a negative attitude. They were not willing to work from the bottom up, especially given the poor working conditions in the industry. Employers emphasised that personality and attitude were the most important attributes. Employers also believed that students do not consider that this applies to students. In general, employers' expectations were unmet, resulting in a dilemma between employers and students.
